# Host‐Associated Bacterial Community Changes After Laboratory Introduction Vary With *Wolbachia* Presence

**DOI:** 10.1111/1462-2920.70265

**Published:** 2026-03-15

**Authors:** Pina Brinker, Joana Falcao Salles, Leo W. Beukeboom, Michael C. Fontaine

**Affiliations:** ^1^ Groningen Institute for Evolutionary Life Sciences (GELIFES) University of Groningen Groningen the Netherlands; ^2^ Institute of Evolutionary Ecology University of Regensburg Regensburg Germany; ^3^ MIVEGEC, Univ. Montpellier, CNRS, IRD Montpellier France

**Keywords:** *Asobara japonica*, community analyses, laboratory studies, longitudinal study, symbionts

## Abstract

Translocating organisms from their natural habitats to laboratories can significantly alter their microbial communities, yet this impact is often overlooked. While common in research, the effects on microbiomes and how laboratory findings relate to natural field dynamics require further study. Symbionts may stabilise microbial communities or increase susceptibility to change, influencing results. This study investigates the effects of laboratory translocation on host‐microbiome interactions using the parasitic wasp *Asobara japonica* and its endosymbiont *Wolbachia*. Three infected (asexual) and three uninfected (sexual) lines, each with seven iso‐female lines, were introduced into the laboratory to track microbial community changes over four generations via 16S rRNA gene sequencing. Our results show laboratory translocation reduces bacterial diversity, with stochastic processes driving changes in the microbial community. Changes in bacterial composition differed between sexual and asexual lines. Over four generations, the asexual wasps' bacterial community became more similar, while sexual wasps exhibited greater diversity. Notably, changes in bacterial communities emerged over generations rather than in the first generation. Finally, *Wolbachia* abundance varied following laboratory introduction, likely impacting bacterial community structure and assembly over time. Overall, our research highlights how laboratory conditions can affect host‐associated microbial communities in different ways, potentially impacting their functions and host interactions.

## Graphical abstract

The endosymbiont *Wolbachia* promotes homogenisation and delays the microbial community response of the parasitic wasp *Asobara japonica* after a significant environmental change. This suggests that key microbial taxa can mediate community shifts and has important implications for experimental design in microbial ecology, particularly in translating lab findings to natural systems.

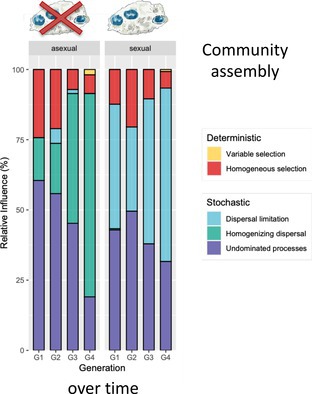

## 1 Introduction

The host associated microbial community, the microbiome, is an essential component of all organisms and is shaped by intricate interactions with the host, among microbial members, and environmental conditions (Leibold et al. 2004; Adair and Douglas 2017; Brinker et al. 2019, 2023; Uren Webster et al. 2020). This community and its interactions are sensitive to disturbances and, if disrupted, can, for example, in insects, lead to starvation (Hosokawa et al. 2010), susceptibility to parasites (Dheilly et al. 2017), or undermine disease resistance (Dacey and Chain 2020). Moreover, disruptions not only have the potential to alter the impact of symbionts on both the host and its associated microbial community (Bénard et al. 2020) but can extend to the entire ecosystem within which the host resides (Pita et al. 2018; Schapheer et al. 2021).

Disruptions of host‐associated microbial communities and their interactions are often caused by environmental changes (de Vries et al. 2004; Russell and Moran 2006; Ochman et al. 2010; Colman et al. 2012; Ferguson et al. 2018; Duan et al. 2020). One peculiar and substantial environmental change is the translocation of organisms from nature into the laboratory or *vice versa* (Gall et al. 2017; Waltmann et al. 2019). The translocation of an organism can lead to changes in the diversity and abundance of microbial communities associated with the organism and thus influence essential traits of the relocated host. After the translocation from the wild into the laboratory, various environmental factors experienced by a host are likely to change, with missing fluctuations in temperature, humidity and other factors, as well as changes in diet likely having percolating effects on the host‐associated microbial community (Ochman et al. 2010; Colman et al. 2012; Luo et al. 2021). Moreover, the translocation from the wild into the laboratory has the potential to influence the horizontal acquisition of free‐living microbes present in water, soil or air and microbes from other species, which can negatively affect hosts if they rely on environmental acquisition or horizontal transmission of microbes (Pons et al. 2019; Acevedo et al. 2021). Finally, from a host perspective, individuals may experience a decrease in population size and a reduction in genetic diversity, potentially leading to changes in the microbial community (Smith et al. 2015; Brinker et al. 2023). Translocations of organisms and concomitant environmental changes are not only relevant for studies focusing on understanding symbiotic interactions (Brinker et al. 2019) but additionally carry economic implications, given the increasing commercial breeding of organisms for purposes such as feed and food production (Francuski and Beukeboom 2020) or their deployment as biological agents for pest control (Parra and Coelho 2022) and disease management (Moreira et al. 2009; King et al. 2018). Finally, with anthropogenic activities leading to drastic environmental changes and microbiome research relying on experimental work in laboratories and the translocation of organisms from nature into the laboratory and *vice versa*, which inherently involve environmental alterations, an understanding of host‐associated microbial community reactions to environmental changes during or after translocation will become more and more important.

We know that individuals reared in the laboratory harbour less diverse microbial communities compared to those living in the wild. However, the changes in microbial communities after laboratory introduction need to be evaluated to determine whether they occur randomly (stochastically) or follow a pattern (deterministically), which could inform future modelling approaches that predict such changes. Additionally, it's important to investigate if changes occur differently in systems in which a powerful symbiont is present, as symbionts could act as a stabiliser of the microbial community (Herren and McMahon 2018) or make them more susceptible to changes. To investigate this, we will use a simple model system represented by the haplodiploid parasitic wasp *Asobara japonica*. Like other insects, *A. japonica* has a relatively low microbial diversity (Engel and Moran 2013; Brinker et al. 2023) and naturally occurs with and without the endosymbiotic bacterium *Wolbachia*, whose presence modulates host reproduction. *Wolbachia* infected wasps reproduce asexually, producing only females from unfertilised eggs (thelytokous parthenogenesis; Kremer et al. 2009), whereas uninfected wasps reproduce sexually (arrhenotoky). A comparison between infected and uninfected wasp lines thus allows us to infer whether the presence of symbionts influences patterns of microbial community change associated with translocations through microbe‐microbe interactions (Brinker et al. 2019). In addition, the use of three sexual (uninfected) and three asexual (infected) distinct genetic lines of *A. japonica* (Brinker et al. 2023) allows us to infer whether patterns depend on host genetic background, population structure, and microbiome composition, based on the whole body. We first evaluate changes in the wasps' associated bacterial communities across four generations after their transfer from nature to the laboratory. We anticipate changes in both infected and uninfected wasps, with the most significant shifts in the initial generations due to substantial environmental changes after translocation. We also explore how *Wolbachia*, which is confounded by reproductive mode and geography, might influence these changes. We hypothesise that infected (asexual) wasps will likely exhibit fewer microbial community changes, thanks to potential community‐stabilising effects (Herren and McMahon 2018) and a lack of genetic diversity loss from asexual reproduction. In contrast, sexual wasps not only lack *Wolbachia* as a potential stabilising key bacterial taxon but might also experience a loss of genetic variability due to laboratory rearing introduced bottlenecks. Therefore, we expect the microbial community changes of sexual wasps may be more substantial than those of asexual wasps. Finally, we investigate whether deterministic or stochastic processes govern bacterial community changes upon laboratory introduction, aiming to discern predictability patterns in these shifts.

## 2 Material and Methods

### 2.1 Wasp Collection and Rearing

**FIGURE 1:**
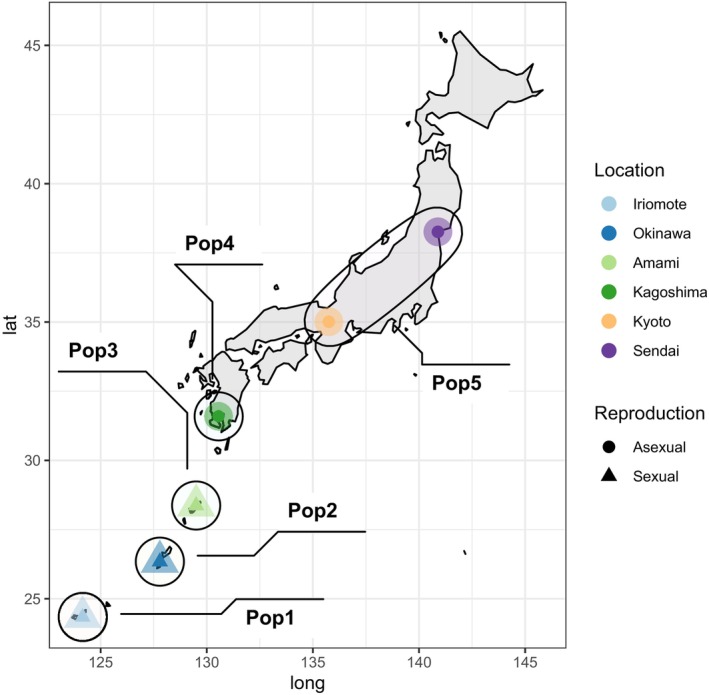
Schematic map of Japan showing collection sites of *Asobara japonica* used to establish laboratory lines in this study. Locations with sexually reproducing wasps in the south of Japan are indicated by a triangle and locations with asexually reproducing wasps in the north of Japan by a circle. Black ellipses show the population identity of wasps collected from the location inferred from population genetic analyses (see Brinker et al. 2023).

*Asobara japonica*, a larval parasitoid of various *Drosophila* species native to southeastern Asia, occurs naturally infected with *Wolbachia*, causing asexual (thelytokous parthenogenetic) reproduction on Japan's main island and uninfected, reproducing sexually on the southern islands of Japan (Mitsui et al. 2007). For this study, wasps from six locations (three infected with *Wolbachia*, three uninfected; see Figure 1) were collected in June 2017 as described in Brinker et al. (2023). In brief, traps containing smashed banana and yeast were set outside at different locations for 6–7 days to attract fruit flies (*Drosophila* spp.) to lay eggs, subsequently attracting parasitic wasps to oviposit in their larvae. Baits were collected after 6–7 days, and developing *Drosophila* larvae were separated from the bait material and placed in tubes containing a layer of agar. Tubes were brought to the laboratory in the Netherlands and stored in an incubator at 25°C with a light–dark cycle of 16 h light and 8 h darkness (LD16:08) until wasps hatched (approximately 19–22 days). Emerging wasps were identified to species level based on morphological features (Guerrieri et al. 2016; Mitsui et al. 2007). Each of the collection locations represents a genetically distinct population (Sexual Pop 1—Pop 3, Asexual Pop 4), except the two most northern ones, Kyoto and Sendai, which belong to the same population (Pop 5) (Brinker et al. 2023). Asexual populations occur in different environmental conditions, with subtropical conditions in Kagoshima (Pop 4) and more temperate conditions in Kyoto and Sendai (both Pop 5). In contrast, all sexual locations have a subtropical climate.

As described, wasps were collected as larvae and brought to the laboratory in the Netherlands, where adults hatched. This way, we ensured a natural starting point for the subsequent laboratory rearing. From these G0 mother wasps, we created seven replicated iso‐female lines from each location (total *n* = 42, per location = 7) using randomly selected hatched female wasps. Lines were maintained for another four generations under stable laboratory conditions (total *n* = 168, per location *n* = 28, i.e., four generations times seven lines). Each new generation was started by placing three to four randomly selected females (offspring of the previous generation, matured for 3 days during which they were fed honey) into bottles containing agar coated with a thin layer of yeast (AB Mauri S.p.A., Italy) as well as second instar *D. melanogaster* (ww‐strain) larvae. These bottles were maintained under stable laboratory conditions (25°C, LD16:08 light cycle) throughout the experiment. For the sexually reproducing wasps, two to three randomly selected males were added to each bottle to secure mating of females. Ten females per replicate, that is, the seven replicate iso‐female lines per location, were pooled and frozen at −80°C for microbial DNA extraction in each generation, resulting in seven coherent replicates per location and generation (total *n* = 168, per location *n* = 28).

### 2.2 Bacterial DNA Extraction

For bacterial DNA extraction, 10 pooled wasps were first washed to remove any environmental contamination (1 min in 70% ethanol and 3× in sterile water). We created a pool of wasps and extracted whole bodies to represent variation within the iso‐female lines and the entire bacterial community of the wasps. DNA was then extracted and purified using the DNeasy Power Soil DNA Isolation Kit, following the manufacturer's protocol (Power Soil, MoBio Laboratories Inc., California, United States), except that wasps were snap‐frozen in liquid nitrogen and crushed with a sterile pestle in a 1.5 mL tube before being added to the homogenisation tubes and homogenised for 15 min using a grinder (Kaiser, Germany). After extraction, DNA was eluted in 100 μL C6 solution provided by the manufacturer and stored at −20°C for further processing and Illumina MiSeq sequencing (2 × 300 bp MiSeq, ~40,000 reads/sample) of the V4 region of the bacterial 16S rRNA gene to the Minnesota genomic centre (Gohl et al. 2016).

### 2.3 Bacterial Community Analyses

Demultiplexed 300 bp paired‐end reads were processed following the *dada2* pipeline v.1.18.0 (Callahan et al. 2016) in *R* v. 4.0.2 (R Core Team 2020). First, read quality was checked by visualising the quality score. Positions with a lower mean quality score than 30 were removed. The first 10 nucleotides of forward and reverse reads were trimmed, and the forward reads truncated to 250, and the reverse reads to 230 bases. Next, reads were dereplicated, and expected errors were removed. This was followed by merging of the reads and removal of chimaera errors. Finally, taxonomy was assigned using the pre‐trained Silva 138 taxonomy classifier (McLaren and Callahan 2021), creating an amplicon sequence variants (ASV) table and an unrooted neighbour‐joining tree using the *phangorn* v.2.7.1 R package (Schliep 2011; Schliep et al. 2017). These output files were then used to create a *phyloseq* object (R package *phyloseq* v.1.34; McMurdie and Holmes 2013). Sequences identified as chloroplast, mitochondria, archaea, or uncharacterised reads at the phylum level, and three outliers based on DNA quality (Sexual: A24 G4; Asexual: Ky2 G2; Ky19 G3) were removed from this object. The dataset was rarefied to 1196 reads and will be referred hereafter as the “full dataset.” From the full dataset, a second *phyloseq* object was created in which reads belonging to the genus *Wolbachia* were removed. Taxa abundance of the *phyloseq* object without *Wolbachia* reads was normalised following the *edgeR* method (*microbiomeSeq* v.0.1 R package; Ssekagiri 2020) prior to ordination analyses and will be referred to as “reduced dataset” hereafter. Moreover, to investigate the effect of laboratory introduction on the total endosymbiont itself, *Wolbachia* reads were extracted from the normalised (*edgeR* method, *microbiomeSeq* v.0.1 R package; Ssekagiri 2020) unrarefied dataset to create a *Wolbachia* read count table.

Statistical analyses were performed in *R* v.4.0.2 (R Core Team 2020). All analyses comparing patterns in asexual vs sexual lines were run with the full and reduced datasets to infer the effect of the high abundance of *Wolbachia* reads in the asexual samples on the analyses. Alpha diversity estimates (i.e., observed number of ASV and Shannon diversity) were calculated with the *phyloseq* function “*estimate_richness*.” Diversity estimates were analysed in separate linear mixed‐effects models (LMM, R package “*lme4*”; Bates et al. 2015) with generation (G1 to G4), reproductive mode (asexual, sexual), and their interactions as fixed predictors and location as a random effect. Similarly, the *Wolbachia* read counts were analysed in separate linear mixed‐effects models (LMM, package “*lme4*”; Bates et al. 2015) with generation (G1 to G4) as fixed predictor and location as a random effect. To assess the significance of the predictor generation, this model was compared to a null (intercept only) using a likelihood ratio test (LRT). To assess the significance of predictors, models were compared to null (intercept only) or reduced models using LRT. Model assumptions were checked with model diagnostic tests and plots implemented in the package *DHARMa* v.0.4.4 (Hartig 2021). As reproductive mode depended on generation for observed ASV number (significant interaction), we created two subsets separating asexual and sexual data for further testing. Pairwise comparisons between factor levels of a significant predictor in these models were performed using Tukey post hoc tests adjusting the family‐wise error rate according to the method of Westfall (package *multcomp*; Hothorn et al. 2008). Additionally, the effect of generation on observed ASV number and Shannon diversity, as well as the effect of generation on *Wolbachia* read counts, was analysed separately for each location using an ANOVA followed by pairwise comparisons for the reproductive modes. This was done considering each location as a different host genetic background, as each location is a genetically distinct population (except Kyoto and Sendai, which belong to the same population).

Variation in community composition (beta diversity) among samples was visualised via a principal coordinates analysis (PCoA) based on the Bray–Curtis distance dissimilarity matrices created with the *phyloseq* function ‘*ordinate*’. The number of significant PCo dimensions to interpret was determined based on a visual examination of the scree‐plot (Figures S1 and S2). The effect of reproductive mode (asexual, sexual), lab rearing over the four generations, and location identity, *a.k.a*. genetic background, were tested by performing permutational multivariate analysis of variance using the function ‘*adonis*’ and 999 permutations (PERMANOVA) using the v*egan* R package (Anderson and Willis 2003; Oksanen et al. 2016).

Finally, we assessed whether bacterial community assembly is governed by deterministic or stochastic processes, or both and whether these processes are influenced by reproductive mode. For this, we followed the methods outlined in Stegen et al. (2012, 2013). The pairwise phylogenetic turnover between communities was calculated by the mean nearest taxon distance metric (βMNTD) using the ‘*comdistnt’* function (abundance. weighted = TRUE) in ‘*picante’* (Kembel et al. 2010). The difference between the observed βMNTD and the mean of the null distribution was measured in units of standard deviations (of the null distribution), using the convention to *β*‐Nearest Taxon Index (βNTI) (Stegen et al. 2013). βNTI values (derived from pairwise comparisons) were compared between the different generations for both reproductive modes. In brief, βNTI < −2 or > +2 indicates that βMNTD_obs_ deviates from the mean βMNTD_null_ by more than two standard deviations. Thus, βNTI < −2 or > +2 deviate significantly from the model‐based expected phylogenetic turnover, and thus turnover is driven by deterministic processes. Here, βNTI < −2 indicates low turnover (i.e., homogeneous selection), and βNTI > +2 indicates high turnover (i.e., variable selection). Values between −2 and +2 indicate the lack of deviation, implying the dominance of stochastic processes, where community assembly is less influenced by selection and more by chance (Stegen et al. 2012; Dini‐Andreote et al. 2015). Additionally, the Bray–Curtis‐based Raup–Crick metric (Rcbray) was calculated (Stegen et al. 2012, 2013) to partition further the relative influences of stochastic processes. Rcbray > 0.95 indicates dispersal limitation, meaning species movement is restricted, leading to higher community dissimilarity (Zhou and Ning 2017). Rcbray < −0.95 represents homogenising dispersal, meaning high species movement, resulting in decreased community dissimilarity (Zhou and Ning 2017). Rcbray values between −0.95 and 0.95 suggest undominated processes when neither selection nor dispersal dominates community assembly (Dini‐Andreote et al. 2015).

## 3 Results

### 3.1 Bacterial Diversity Changes Over Generations

Bacterial community diversity in *Asobara japonica* was assessed across three sites for sexual wasps and three sites for *Wolbachia*‐infected asexual wasps over four generations of laboratory culturing. A total of 1203 microbial taxa were identified among 3,779,732 sequence reads. After rarefaction to 1196 reads per sample, 827 taxa were retained, comprising a total of 188,968 reads. Five samples were excluded because they fell below the read threshold set during rarefaction, with one in Kyoto (generation 2), two in Iriomote (generation 2), one in Iriomote (generation 4) and one in Okinawa (generation 4). This led to a total of 163 samples (G1 *n* = 42, G2 = 39, G3 = 42, G4 = 40) used in the following analyses. Upon removing ‘*Wolbachia’* reads, the dataset contained 819 taxa and 114,302 reads.

**FIGURE 2:**
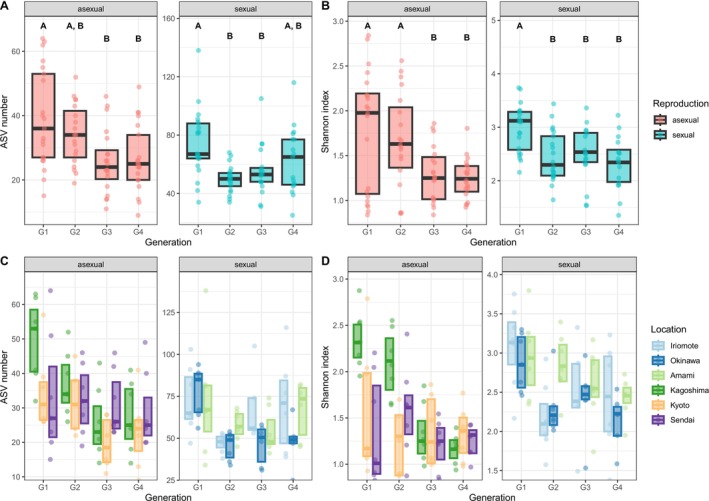
Alpha diversity, expressed as (A) ASV number and (B) Shannon Index, was measured in seven replicated lines of asexual (*Wolbachia*‐infected) and sexual (*Wolbachia*‐uninfected) *A. japonica* wasps over four generations (G1 to G4) in the laboratory. Letters above the boxes indicate significant differences between generations. Alpha diversity was also assessed per location, with (C) ASV number and (D) Shannon Index for seven replicates of three asexual, *Wolbachia*‐infected lines (Kagoshima, Kyoto, Sendai) and three sexual, *Wolbachia*‐uninfected lines (Iriomote, Okinawa, Amami) over four generations (G1 to G4) in the laboratory. Only the locations Okinawa and Kagoshima showed statistically significant differences over generations (see Table S1). Boxplots show the median and interquartile range.

Sexual wasps in which *Wolbachia* is absent exhibited a higher species diversity, in terms of observed ASV number as well as Shannon diversity, than the asexual wasps. However, in both sexual and asexual wasps, this diversity decreased over the four generations of laboratory culturing (Figure 2A,B). This decrease was dependent on the reproductive mode for the number of bacterial species (LMM; interaction generation × reproductive mode; LRT, *χ* ^2^ = 10.94, df = 3, *p* = 0.012), but not for Shannon diversity (LMM; interaction generation × reproductive mode; LRT, *χ* ^2^ = 5.92, df = 3, *p* = 0.116). To delve deeper into the effect of alpha diversity changes (observed ASV number and Shannon diversity) across generations, we separated the data into the two reproductive modes: sexual and asexual lines. For both reproductive modes, observed ASV number and Shannon diversity (tested separately) were significantly affected by generation (Figure 2A,B; sexual lines: observed ASV number: LRT, *χ* ^2^ = 18.42, df = 3, *p* < 0.001; Shannon: LRT, *χ* ^2^ = 19.28, df = 3, *p* < 0.001; asexual lines: observed ASV number: LRT, *χ* ^2^ = 18.31, df = 3, *p* < 0.001; Shannon: LRT, *χ* ^2^ = 19.66, df = 3, *p* < 0.001). However, there were differences in the rate of change over generations between sexual and asexual lines. Sexual lines showed a reduction in alpha diversity (observed ASV number and Shannon diversity) already after the first generation (Figure 2A,B, Table S1). In contrast, asexual lines showed a reduction in diversity only after the second generation (Figure 2A,B, Table S1). Further examination of this generation effect for each wasp line revealed that these patterns were driven by one line for both reproductive modes: Okinawa for sexual and Kagoshima for asexual reproducing wasps, respectively (see Figure 2C,D, Table S1). The Okinawa line (Pop 2) showed a significant decrease in alpha diversity (ANOVA: observed ASV number *F*_3,21_ = 10.26, *p* < 0.001; Shannon *F*_3,21_ = 4.582, *p* = 0.013) after the first generation and the Kagoshima line (Pop 4), at generation 3 (ANOVA: observed ASV number *F*_3,24_ = 7.72, *p* < 0.001; Shannon *F*_3,24_ = 25.86, *p* < 0.001; Table S1).

Removal of *Wolbachia* reads from the dataset did not change these patterns (Figure S3, Table S1), with the exception that the pattern for Shannon diversity changed in asexual wasps. It became more similar to the pattern observed in sexual wasps, that is, a reduction occurred already after the first generation. The reduced dataset revealed a significant interaction between generation and reproductive mode for observed ASV number (LRT, *χ* ^2^ = 10.74, df = 3, *p* = 0.013) but not for Shannon diversity (LRT, *χ* ^2^ = 0.42, df = 3, *p* = 0.74). To maintain comparability between the full and reduced datasets, we again examined the effect of alpha diversity changes (observed ASV number and Shannon diversity) over generations separately for each reproductive mode. For sexual wasps, observed ASV number and Shannon diversity were significantly influenced by generation (Figure S3; Observed ASV number: LRT, *χ* ^2^ = 19.049, df = 3, *p* < 0.001; Shannon: LRT, *χ* ^2^ = 20.05, df = 3, *p* < 0.001), with generation one differing significantly from the other three generations (Figure S3, Table S1). Also, in asexual wasps, observed ASV number and Shannon diversity were still significantly influenced by generation (Figure S3; observed ASV number: LRT, *χ* ^2^ = 18.14, df = 3, *p* < 0.001; Shannon diversity: LRT, *χ* ^2^ = 13.2, df = 3, *p* < 0.001), with a significant decrease occurring in generation three (Figure S3, Table S1). Investigating this generation effect for each location individually and testing the influence of genetic background, we found that these patterns were driven by two lines, Kagoshima (Pop 4) and Kyoto (Pop 5). The asexual lines showed a significant reduction in observed ASV number (ANOVA: Kagoshima: observed ASV number *F*_3,24_ = 7.67, *p* < 0.001; Kyoto: observed ASV number *F*_3,24_ = 3.35, *p* = 0.003) after the second generation (Figure S4, Table S1). A significant reduction of Shannon diversity was only found for wasps from Kyoto (ANOVA: Shannon *F*_3,24_ = 3.79, *p* = 0.02), which occurred again at generation three (Figure S4, Table S1).

### 3.2 Changes in Bacterial Community Composition Differ Between Reproductive Modes

**FIGURE 3:**
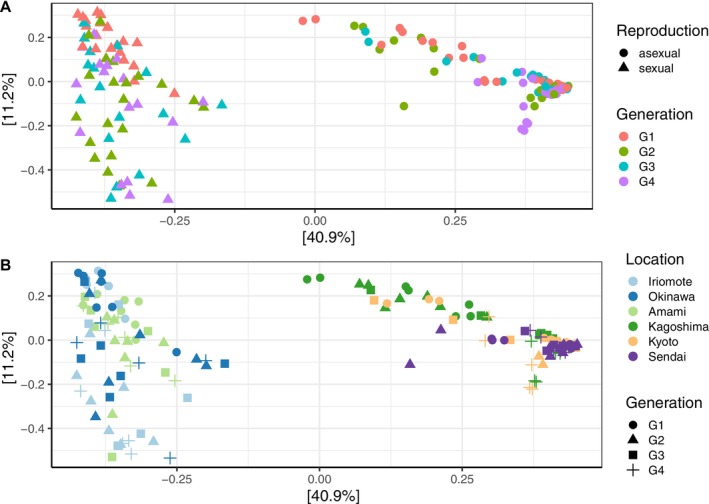
Differences in bacterial community composition between (A) *Wolbachia*‐uninfected, sexual lines (dots) and *Wolbachia*‐infected, asexual lines (triangles) and per (B) location, that is, geographic origin of *A. japonica* over four generations visualised via a principal coordinates analysis (PCoA) based on Bray–Curtis distance dissimilarity matrices. In (A) colours indicate generation and shapes indicate reproductive mode, that is, infection status. In (B) colour indicates location, and shapes indicate generation.

Similar to species diversity, we found distinct differences in the community composition between *Wolbachia* infected (asexual) and uninfected wasps (sexual) (adonis: pseudo‐*F*_1,156_ = 96.67, *R* ^2^ = 0.38, *p* = 0.001), with reproductive mode, explaining 40.5% of the variation and the PCoA showing a clear separation of the sexual and asexual reproducing lines (Figure 3A,B). Moreover, generation explained 11.3% of the variation in the data (adonis: pseudo‐*F*_3,154_ = 2.69, *R* ^2^ = 2.69, *p* = 0.003). The community diversity of asexual wasps became more similar over generations, whereas that of sexual wasps became more distinct, with generation one having the lowest dispersion in sexual lines and asexual lines forming a tight cluster in generations three and four (Figure 3). Considering the genetic background over generations, sexual wasps from Amami (Pop 2) and Okinawa (Pop 3) clustered together. In contrast, the bacterial community composition of sexual wasps from Iriomote (Pop 1) became more variable over generations (Figure 3C,D). For the asexual wasps, lines from Kagoshima (Pop 4) in generations one and two and Kyoto (Pop 5) in generation 1 formed distinct clusters from the other asexual wasps (Figure 3C,D; adonis: pseudo‐*F*_5,152_ = 23.61, *R* ^2^ = 0.44, *p* = 0.001). The reduced dataset, that is, where *Wolbachia* was removed, revealed a similar pattern. Although the clustering for the two reproductive modes became less distinct, it still explained 19% of the variation (adonis: pseudo‐*F*_1,156_ = 12.49, *R* ^2^ = 0.074, *p* = 0.001). Again, generation influenced clustering (adonis: pseudo‐*F*_3,154_ = 3.34, *R* ^2^ = 0.06, *p* = 0.001), explaining 14.1% of the variation. Asexual lines grouped closer together over time, whereas sexual lines became more distinct from each other over generations (Figure S5). Looking at the patterns for single lines, we found that Kagoshima (Pop 4) and Kyoto (Pop 5) had a broader dispersion in the first two generations and became more similar to the community of Sendai (Pop 5), in which the bacterial community composition showed little variation over time (Figure S6). The bacterial diversity of sexual wasps depicted a different pattern and became more diverse with each generation, especially the location Iriomote (Figures S5 and S6; adonis: pseudo‐*F*_5,152_ = 5.64, *R* ^2^ = 0.16, *p* = 0.001).

### 3.3 Number of *Wolbachia* Reads Changes Over Generations

**FIGURE 4:**
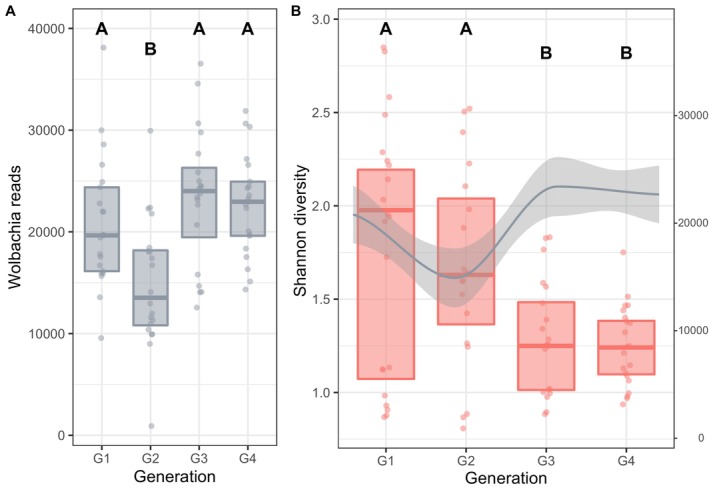
(A) Abundance of *Wolbachia* cells measured as the number of *Wolbachia* sequenced reads in seven replicate lines of three asexual, *Wolbachia*‐infected *A. japonica* populations (Kagoshima, Kyoto, Sendai) over four generations (G1 to G4) in the laboratory. Reads extracted out of the unrarefied but normalised full dataset are plotted. Boxplots show the median and interquartile range. (B) Plot of the Shannon diversity (red boxplots, left *y*‐axis) and a LOESS‐smoother (locally weighted running line smoother) together with confidence intervals on *Wolbachia* reads (grey line and grey area show 95% confidence intervals, right *y*‐axis). Letters above boxes denote statistically significant differences between generations for *Wolbachia* sequence reads (A) and Shannon diversity (B).

We observed a significant fluctuation of *Wolbachia* over generations (Figure 4A; LRT, *χ* ^2^ = 7.395 x 10^−5^, df = 3, *p* < 0.001) with a decrease of endosymbiont abundance at generation 2 (mean *Wolbachia* reads ± S.D. in Table S2A), followed by an increase in generation three to levels similar to generation one, which then stabilised in generation four (mean *Wolbachia* reads ± S.D. in Table S2A; post hoc pairwise comparisons: G2 vs. G3 and G4: all *p* < 0.001; G1 vs. G3 and G4, G4 vs. G3: all *p* > 0.367). These fluctuations of *Wolbachia* do not correlate with the changes in Shannon diversity in generation three (Figure 4B). Moreover, the fluctuations of *Wolbachia* correlate with the homogenisation of the bacterial community composition (beta diversity) in generation three and four in asexual wasps (Figure 3). Investigating the three asexual locations separately, we find that wasps from lines with an initially high number of *Wolbachia* (Pop 5, Kyoto and Sendai) experienced a decrease in the second generation (Figure S7A; mean *Wolbachia* reads ± S.D. in Table S2B; Kyoto ANOVA: *F*_3,22_ = 4.996, *p* = 0.009, post hoc pairwise comparisons: G1 vs. G2, G2 vs. G3 and G4: all *p* < 0.026; all other comparisons: ns; Sendai: ANOVA: *F*_3,24_ = 2.2, *p* = 0.114). In contrast, Kagoshima (Pop 4), which had a comparably lower count of *Wolbachia* in the first generation, showed an increase of *Wolbachia* after the second generation (Figure S7B, mean *Wolbachia* reads ± S.D. in Table S2B; ANOVA: *F*_3,24_ = 4.318, *p* = 0.014, post hoc pairwise comparisons: all *p* > 0.065).

### 3.4 Stochastic Processes Govern Bacterial Community Assembly

**FIGURE 5:**
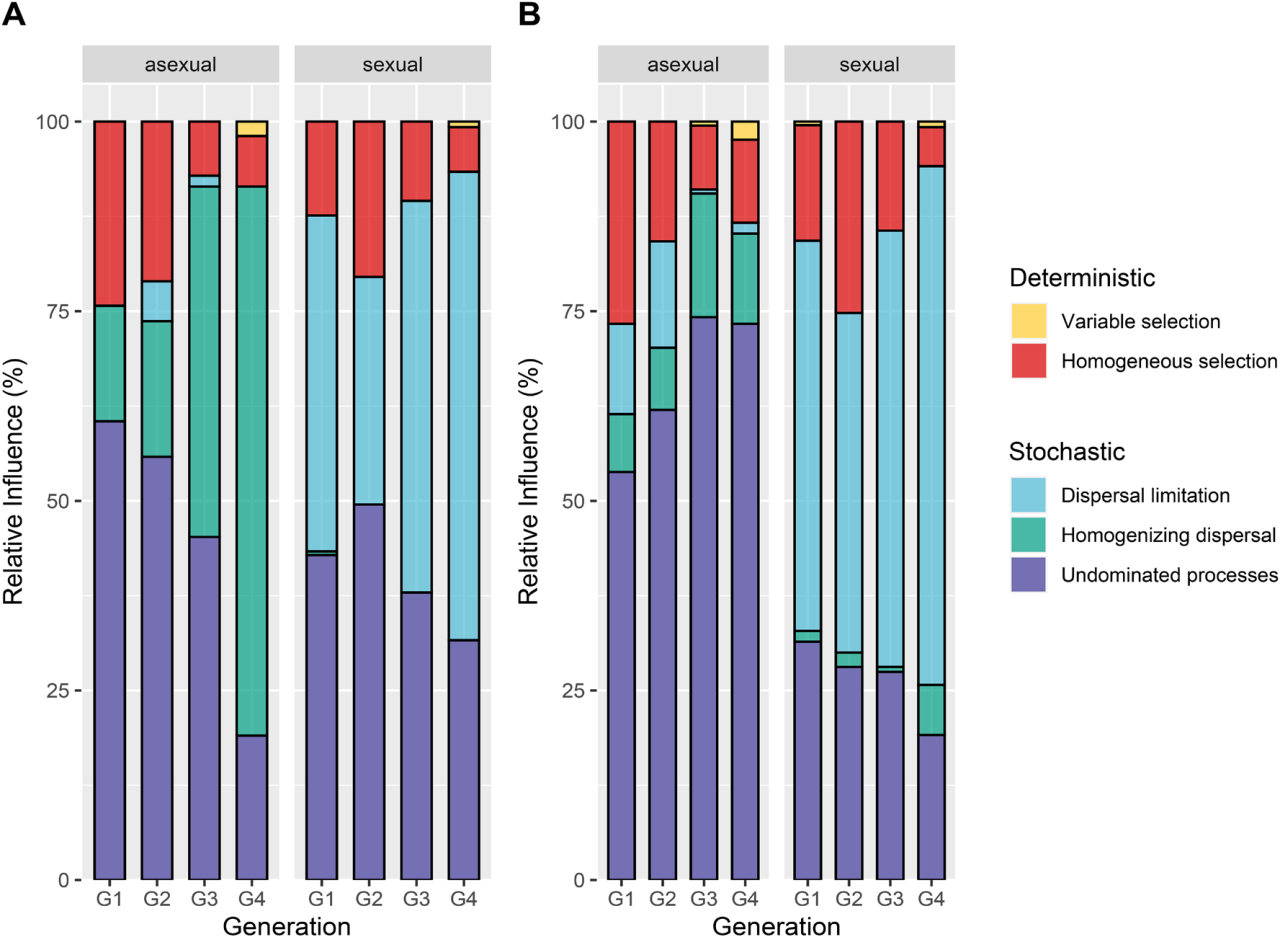
Processes driving bacterial community diversity of seven replicated lines of each asexual (*Wolbachia*‐infected) and sexual (*Wolbachia*‐uninfected) *A. japonica* line reared over four generations (G1 to G4) in the laboratory in (A) the full dataset and (B) after the removal of *Wolbachia* reads. Barplots show the percentage of influencing processes (colours) per generation for the two reproductive modes, asexual (*Wolbachia‐*infected) and sexual (uninfected).

Applying the null model framework of Stegen et al. (2012, 2013), we found that bacterial community assembly over the four generations was mainly governed by stochastic processes (Figure 5; Table S3), such as dispersal limitation—constraint movement of species leading to higher community dissimilarities—homogenising dispersal—high levels species movement lead to more similar communities—(Zhou and Ning 2017) and undominated processes, where it could not be determined which processes were driving changes, that is, neither selection nor dispersal dominated the assembly processes (Jia et al. 2020).

Deterministic effects occurred for both reproductive modes (homogeneous selection) and decreased over time (Table S3). In the asexual lines, homogenising dispersal dominated community assembly, next to undominated processes. After generation two, homogenising dispersal was the dominant driver (Figure 5A; Table S3). Removal of *Wolbachia* reads from the data decreased the influence of stochastic processes (homogenising dispersal) on community changes over undominated processes (Figure 5B). For the sexual lines, dispersal limitation was one of the most critical stochastic processes driving the assembly of bacterial communities, increasing in relative importance over the generations.

## 4 Discussion

Understanding how microbial communities will react to environmental changes is important, given the increasing disturbances of natural habitats by anthropogenic activities. Studying how wild populations change following transfer to controlled laboratory conditions is one way of gaining insight into this. Moreover, investigations of the importance of the microbiome for host fitness, symbiont research and economic rearing often require laboratory experiments in which stable environmental conditions—that is, a reduced mating pool, and limited scope for horizontal transmission of microbes—induce changes in the host's microbiome. Here, we investigated the effect of laboratory introduction on bacterial communities associated with an insect host depending on host geographic origin, genetic background (population structure), and the presence of the endosymbiont *Wolbachia*, which modulates host reproduction. As expected, laboratory rearing imposed a reduction in host‐associated bacterial diversity. Interestingly, this reduction differed between host reproductive modes (symbiont presence) regarding factors driving the change and timing. This suggests that a powerful symbiont like *Wolbachia*, likely in combination with the host genetic background, plays a significant role in steering alterations in bacterial communities when environmental conditions undergo a shift.

As anticipated, the transition of wasps from their natural habitat into a laboratory setting resulted in decreased bacterial diversity and altered community composition in both sexual and asexual wasps. Indeed, multiple studies found that species reared in the laboratory harbour less diverse microbial communities than their natural counterparts (Gall et al. 2017; Waltmann et al. 2019; Brown et al. 2023). This reduction is likely due to the loss of environmentally obtained microbes from resources such as food and free‐living microbes from their environment (Pons et al. 2019; Acevedo et al. 2021; Luo et al. 2021). Interestingly, in our study, this reduction only occurred after the second and third generations for sexual and asexual wasps, respectively. This indicates a maternal transmission of microbes from G0 mothers, who grew up in nature, to their offspring (G1), hatched in the laboratory. Such maternal transmission is well‐known in many organisms (Funkhouser and Bordenstein 2013) but has not been described in *A. japonica* . However, as we did not screen G0 mothers, this awaits further verification.

Our findings also indicate that *Wolbachia* impacted how bacterial communities changed. The delayed reduction in alpha diversity and the gradual homogenisation of the bacterial community composition (beta diversity) in asexual wasps suggests that *Wolbachia* not only shapes the bacterial community of its host but potentially acts as a bacterial community stabiliser (Herren and McMahon 2018). A similar influence of *Wolbachia* has also been found in fruit flies ( *Drosophila melanogaster* ) (Simhadri et al. 2017), the small brown planthopper ( *Laodelphax striatellus* ) (Duan et al. 2020), and artificially infected mosquito adults ( *Aedes aegypti* ) (Audsley et al. 2018). Therefore, the results of our study indicate that the presence of *Wolbachia* may buffer a potential influence of environmental factors affecting the bacterial community of a host (de Vries et al. 2004; Russell and Moran 2006; Ochman et al. 2010; Colman et al. 2012; Ferguson et al. 2018; Duan et al. 2020).

We also found that the endosymbiont *Wolbachia* itself is influenced by laboratory introduction. Over four generations, the abundance of *Wolbachia* reads changed to uniformly high levels of *Wolbachia* in wasps from all three asexual lines by the fourth generation. Similar changes in *Wolbachia* were found in *Tetranychus* mites, where some mites experienced an increase in *Wolbachia* titre after laboratory introduction (Zélé et al. 2020). Interestingly, our findings indicate that these *Wolbachia* fluctuations are linked to the initial levels of the bacterium. The two northern asexual wasp lines, Kyoto and Sendai (both Pop 5), which initially had a high quantity of *Wolbachia* reads, saw a decline in the endosymbiont after transitioning, followed by a rebound to their initial levels. Conversely, the more southern and genetically distinct Kagoshima population, which initially had a relatively low number of *Wolbachia*, saw an increase in *Wolbachia* over time. The reasons for these initially different *Wolbachia* abundances in the three populations are unknown. However, the subtropical conditions at Kagoshima may have influenced *Wolbachia* abundance as symbionts appear to be sensitive to high temperature (Van Opijnen and Breeuwer 1999; Hurst et al. 2000; Corbin et al. 2017; Sumi et al. 2017) and show a general geographical pattern of lower prevalence in warmer regions (Corbin et al. 2017). Therefore, the more temperate temperatures in the laboratory could have caused an increase.

The observed *Wolbachia* fluctuations might have contributed to the decline in bacterial diversity and the shift in microbial community composition by the third generation observed in our data. Potentially, the reduction in *Wolbachia* numbers at generation two of the two northern lines (Pop 5: Kyoto and Sendai) led to a significant decrease in alpha diversity in their third generation. This may indicate that the stabilising effect of *Wolbachia* was weakened in generation two due to its low abundance. In other words, the impact of *Wolbachia* may not have been strong enough to maintain its control on the bacterial community composition for the next generation. Moreover, the equal abundance of *Wolbachia* in all samples at generation 4 could have been a driver of the homogenisation of the bacterial community composition (beta diversity) of asexual wasps at generation 4. This suggests that symbiont density within an organism is crucial for sustaining symbiont effects, as observed in the correlation between *Wolbachia* density and its protection against viruses in *Drosophila simulans* (Martinez‐Sañudo et al. 2018). Furthermore, in our study system, *A. japonica* , asexual reproduction relies on a *Wolbachia* threshold (Ma et al. 2015), where *Wolbachia* numbers below a certain threshold result in unsuccessful host reproductive manipulation. Taken together, we find that *Wolbachia* is affected by the introduction into the laboratory with potential knock‐on effects on the host‐associated microbial community, a decrease in alpha diversity, and a homogenisation of the microbial community composition over time.

Lastly, using multiple replicates per line, we found that stochastic processes mainly drove bacterial community changes for both reproductive modes. This aligns with the theory that host‐microbiome variation is predominantly driven by stochastic processes (Adair and Douglas 2017), as indicated by various studies on community assembly (see Obadia et al. 2017; Vega and Gore 2017; Sieber et al. 2019; Brown et al. 2020; Lou et al. 2021). Interestingly, although stochastic processes mainly drove bacterial community changes, we found differences in the processes that dominate changes over generations between the two reproductive modes. In asexual wasps, the process of homogenising dispersal became more dominant over time, likely because the bacterial community became more similar. In contrast, the process of dispersal limitation increased over time in sexual wasps, probably reflecting the higher dissimilarities of the bacterial communities associated with the sexual lines over time. The fact that wasps reacted differently depending on symbiont presence or absence suggests that other factors, apart from environmental factors, must influence bacterial community changes, as all wasps were reared under the same conditions. One of these factors may be the host's genetic background. Sexually reproducing wasps will likely experience a loss of genetic diversity when introduced to the laboratory due to the reduced genetic mating pool and adaptation to laboratory conditions. This may influence the bacterial community in turn. Indeed, it is known that host genotypes can influence microbial composition, with communities following host phylogenetic signals (Kolasa et al. 2019; Lim and Bordenstein 2020) and having population‐specific microbiomes (Bouchon et al. 2016; Falony et al. 2016; Brinker et al. 2019; Rudman et al. 2019). Although we did not find clear patterns associated with population structure for alpha and beta diversity of asexual wasps, we cannot exclude that the influence of *Wolbachia* may have overpowered such a signal.

## 5 Conclusion

Here, we observed that microbial communities respond differently to environmental changes depending on whether a symbiont is present. Wasps carrying the endosymbiont *Wolbachia* showed a slower decrease in diversity compared to uninfected wasps. Moreover, this reduction in diversity does not always lead to homogenisation of the communities, as found for *Wolbachia* infected wasp lines, but can sometimes promote diversification, as seen for uninfected wasps. This suggests that the presence of symbionts with strong host effects, such as *Wolbachia*, may influence the timing and nature of such changes. Moreover, we also found that introducing microbes in the laboratory tends to reduce host microbial diversity, which raises concerns about the representativeness of lab‐reared individuals regarding natural microbiota profiles. Consequently, results obtained under laboratory conditions concerning microbiota composition, function, and host interactions might not fully reflect what occurs in nature. All this highlights the need to bridge the gap from laboratory findings to the effect in the wild. This is a general challenge for researchers and is currently discussed in plant‐focused (Sergaki et al. 2018; Sessitsch et al. 2019) and human‐based microbial research (Amato et al. 2019) and should be applied to more research fields.

## Author Contributions

**Pina Brinker:** conceptualization, data curation, formal analysis, methodology, writing – review and editing, funding acquisition, investigation, validation, visualization, writing – original draft. **Joana Falcao Salles:** funding acquisition, writing – review and editing, validation. **Leo W. Beukeboom:** funding acquisition, writing – review and editing, resources. **Michael C. Fontaine:** validation, funding acquisition, writing – review and editing.

## Funding

This work was supported by Adaptive Life scholarship of the University of Groningen, The Netherlands.

## Ethics Statement

All research described in this manuscript was conducted in accordance with the ethical standards of *Environmental Microbiology* and relevant institutional and national guidelines. No human or animal subjects were involved.

## Conflicts of Interest

The authors declare no conflicts of interest.

## Supporting information


**Data S1:** Supporting Information.

## Data Availability

Metadata and scripts are available on figshare (https://figshare.com/s/54bcd676a21d53add564). Additionally, 16S rRNA data with sample information have been deposited in NCBI's SRA archives under BioProject ID PRJNA1272023 (accession numbers SAMN48891735‐SAMN48891899).
