# Supplementary material for: Host‐Associated Bacterial Community Changes After Laboratory Introduction Vary With Wolbachia Presence
Source: Environ Microbiol. 2026 Mar 15;28(3):e70265. doi: 10.1111/1462-2920.70265 (PMC12989319; doi:10.1111/1462-2920.70265)
Supplement: Supplementary file 1 — Data S1: Supporting Information. [file EMI-28-e70265-s001.pdf]

Supplementary information for

Host-associated bacterial community changes after laboratory introduction vary with Wolbachia presence.

Running title: Symbiont-influenced microbiome assembly

Pina Brinker[1,2*§], Joana Falcao Salles[1¥], Leo W. Beukeboom [1$], Michael C. Fontaine[1,3#]

[1] Groningen Institute for Evolutionary Life Sciences (GELIFES), University of Groningen, The Netherlands

[2] Institute of Evolutionary Ecology, University of Regensburg, Regensburg, Germany

[3] MIVEGEC, Univ. Montpellier, CNRS, IRD, Montpellier, France

[§]ORCID number: https://orcid.org/0000-0003-4723-4077
[¥]ORCID number: https://orcid.org/0000-0003-4317-7263
[$]ORCID number: https://orcid.org/0000-0001-9838-9314
[#]ORCID number: https://orcid.org/0000-0003-1156-4154

*Correspondence: pina.brinker@ur.de (Pina Brinker)

This PDF file includes:

Table S1-S3

Figures S1-S7

Tables

Table S1: Statistical output of alpha diversity (ASV number and Shannon diversity) analyses for *Wolbachia*-uninfected, sexual (Iriomote, Okinawa, Amami) and *Wolbachia*-infected asexual (Kagoshima, Kyoto, Sendai) lines separately per line (seven replicates per location) once for the full dataset and once for the reduced dataset with *Wolbachia* reads removed. Given are degrees of freedom (DF), F-value (F) and p-value (pVal) of the likelihood-ratio test (LRT) together with the pairwise comparisons between generations (G1 to G4) using Tukey post-hoc test. Sqrt indicates that data needed to be square root transformed.

Diversity	Location	DF	F	pValue	G1 vs G2	G1 vs G3	G1 vs G4	G2 vs G3	G2 vs G4	G3 vs G4
ASV number	Iriomote	3	2.36	0.1	NS	NS	NS	NS	NS	NS
ASV number	Okinawa	3	10.26	<0.001	<0.001	<0.001	0.0012	NS	NS	NS
ASV number	Amami	3	1.195	0.334	NS	NS	NS	NS	NS	NS
ASV number	Kagoshima	3	7.724	<0.001	NS	0.001	0.001	NS	NS	NS
ASV number	Kyoto	3	3.277	0.041	NS	NS	NS	NS	NS	NS
ASV number	Sendai	3	0.144	0.932	NS	NS	NS	NS	NS	NS
Shannon diversiy	Iriomote	3	2.853	0.0618	NS	NS	NS	NS	NS	NS
Shannon diversiy	Okinawa	3	4.582	0.013	0.0268	NS	0.0132	NS	NS	NS
Shannon diversiy	Amami	3	2.111	0.127	NS	NS	NS	NS	NS	NS
Shannon diversiy	Kagoshima	3	25.86	<0.001	NS	<0.001	<0.001	<0.001	<0.001	NS
Shannon diversiy	Kyoto	3	0.563	0.646	NS	NS	NS	NS	NS	NS
Shannon diversiy	Sendai	3	1.051	0.388	NS	NS	NS	NS	NS	NS
	Without *Wolbachia*									
ASV number	Iriomote	3	2.01	0.143	NS	NS	NS	NS	NS	NS
ASV number	Okinawa	3	8.897	<0.001	0.001	0.001	0.003	NS	NS	NS
ASV number	Amami (sqrt)	3	1.395	0.27	NS	NS	NS	NS	NS	NS
ASV number	Kagoshima	3	2.185	0.117	NS	NS	NS	NS	NS	NS
ASV number	Kyoto	3	1.434	0.264	NS	NS	NS	NS	NS	NS
ASV number	Sendai	3	0.736	0.544	NS	NS	NS	NS	NS	NS
Shannon diversiy	Iriomote	3	2.947	0.056	NS	NS	NS	NS	NS	NS
Shannon diversiy	Okinawa	3	4.98	0.009	0.017	NS	0.01	NS	NS	NS
Shannon diversiy	Amami	3	2.4	0.094	NS	NS	NS	NS	NS	NS
Shannon diversiy	Kagoshima	3	2.255	0.109	NS	NS	NS	NS	NS	NS
Shannon diversiy	Kyoto	3	2.553	0.086	NS	NS	NS	NS	NS	NS
Shannon diversiy	Sendai	3	0.487	0.696	NS	NS	NS	NS	NS	NS

Table S2: Mean *Wolbachia* reads and standard deviation per a) generation and b) generation per location for *Wolbachia*-uninfected, sexual (Iriomote, Okinawa, Amami) and *Wolbachia*-infected asexual (Kagoshima, Kyoto, Sendai) lines.

Mean *Wolbachia* reads

(a) per generation

**generation**	**Mean (*SD*)**
G1	10458.05 (11404.41)
G2	7309.02 (8707.42)
G3	12061.51 (12713.55)
G4	11969 (11940.2)

(b) per location and generation

**Location**	Mean (*SD*)			
	**G1**	**G2**	**G3**	**G4**
Iriomote	136.43 (209.06)	67.71 (69.71)	45.17 (18.5)	42.86 (9.03)
Okinawa	45.43 (29.35)	99.57 (127.13)	96.17 (141.14)	249.17 (458.48)
Amami	129.86 (182.39)	53.71 (31.24)	192.43 (361.94)	132.5 (145.98)
Kagoshima	16932.57 (4802.35)	16662.14 (3674.92)	23029 (3777.68)	22698 (5375.22)
Kyoto	22440.14 (4358.91)	10965.5 (6792.35)	20693.67 (8298.72)	22830.71 (5288.67)
Sendai	23063.86 (8341.23)	16527.86 (7461.7)	26119.86 (7384.73)	22495.57 (5002.96)

Table S3: Percentage table of factors driving changes of asexual (Wolbachia-infected) and sexual (Wolbachia-uninfected) *A. japonica* reared in seven replicated lines over four generations (G1 to G4) in the laboratory in A) the full dataset and B) after the removal of Wolbachia reads. Given are the percentages of deterministic factors (variable selection, homogeneous selection) and stochastic driving forces (dispersal limitation, homogenous dispersal, undominated processes) for each generation per reproductive mode.

A

	With *Wolbachia*				
	Variable selection %	Homogeneous selection %	Dispersal limitation %	Homogenizing dispersal %	Undominated processes %
			**Sexuals**		
G1	0.00	12.38	44.29	0.48	42.86
G2	0.00	20.48	30.00	0.00	49.52
G3	0.00	10.46	51.63	0.00	37.91
G4	0.74	5.88	61.76	0.00	31.62
			**Asexual**		
G1	0.00	24.29	0.00	15.24	60.48
G2	0.00	21.05	5.26	17.89	55.79
G3	0.00	7.14	1.43	46.19	45.24
G4	1.90	6.67	0.00	72.38	19.05

B

	No *Wolbachia*				
	Variable selection %	Homogeneous selection %	Dispersal limitation %	Homogenizing dispersal %	Undominated processes %
			**Sexuals**		
G1	0.48	15.24	51.43	1.43	31.43
G2	0.00	25.24	44.76	1.90	28.10
G3	0.00	14.38	57.52	0.65	27.45
G4	0.74	5.15	68.38	6.62	19.12
			**Asexual**		
G1	0.00	26.67	11.90	7.62	53.81
G2	0.00	15.79	14.04	8.19	61.99
G3	0.53	8.42	0.53	16.32	74.21
G4	2.38	10.95	1.43	11.90	73.33

Figures

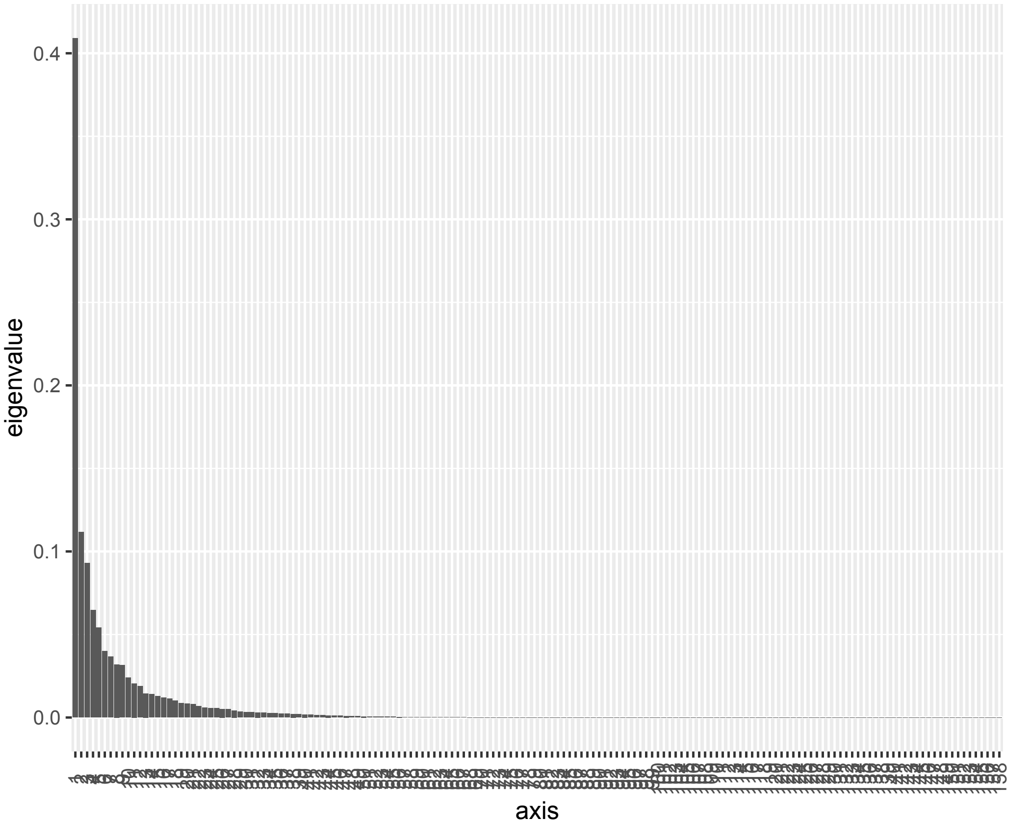

Figure S1: Screen plot of principal components vs eigenvalue for the principal coordinates analysis (PCoA) ordination based on the full dataset's Bray–Curtis distance dissimilarity matrices.

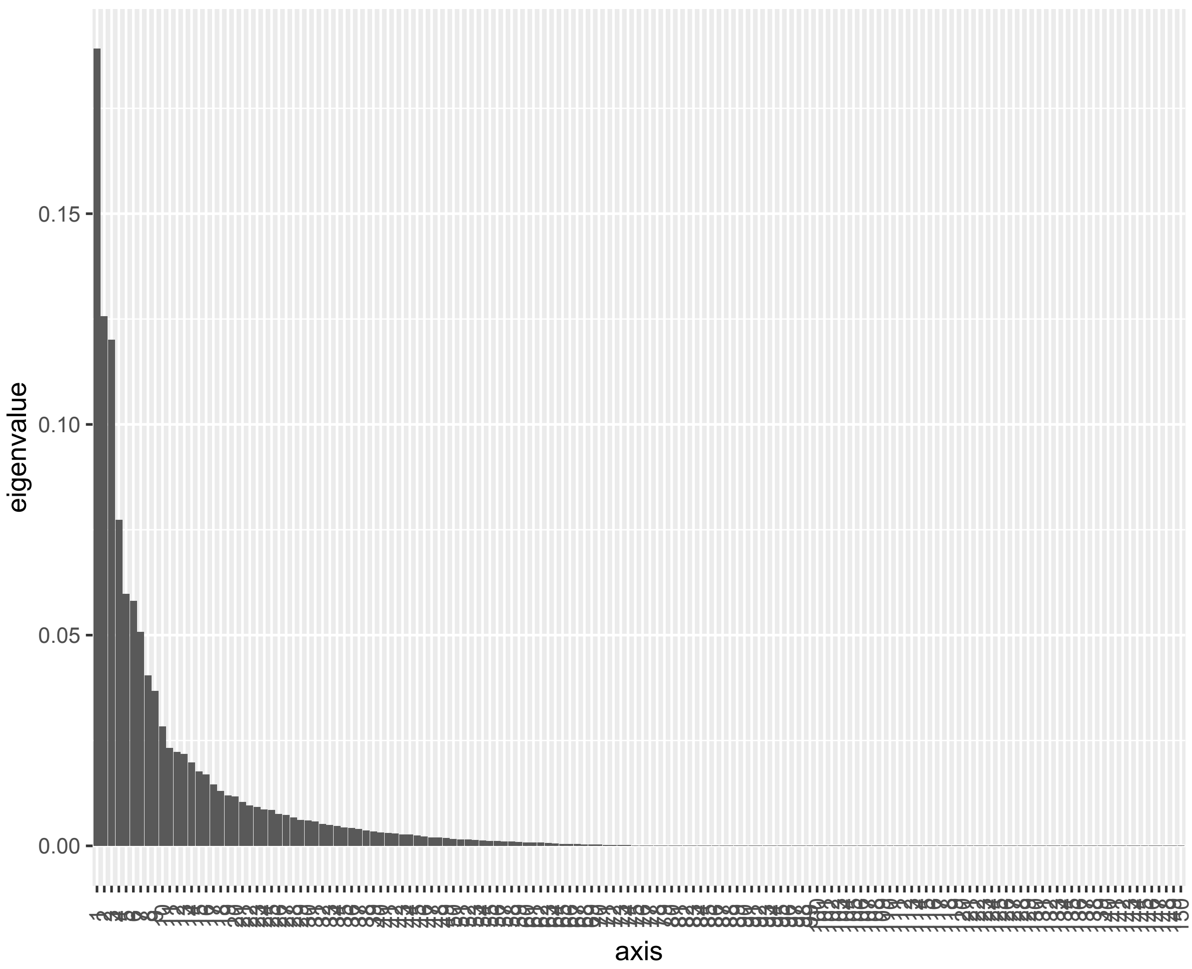

Figure S2: Screen plot of principal components vs eigenvalue for the principal coordinates analysis (PCoA) ordination based on the reduced dataset's Bray–Curtis distance dissimilarity matrices.

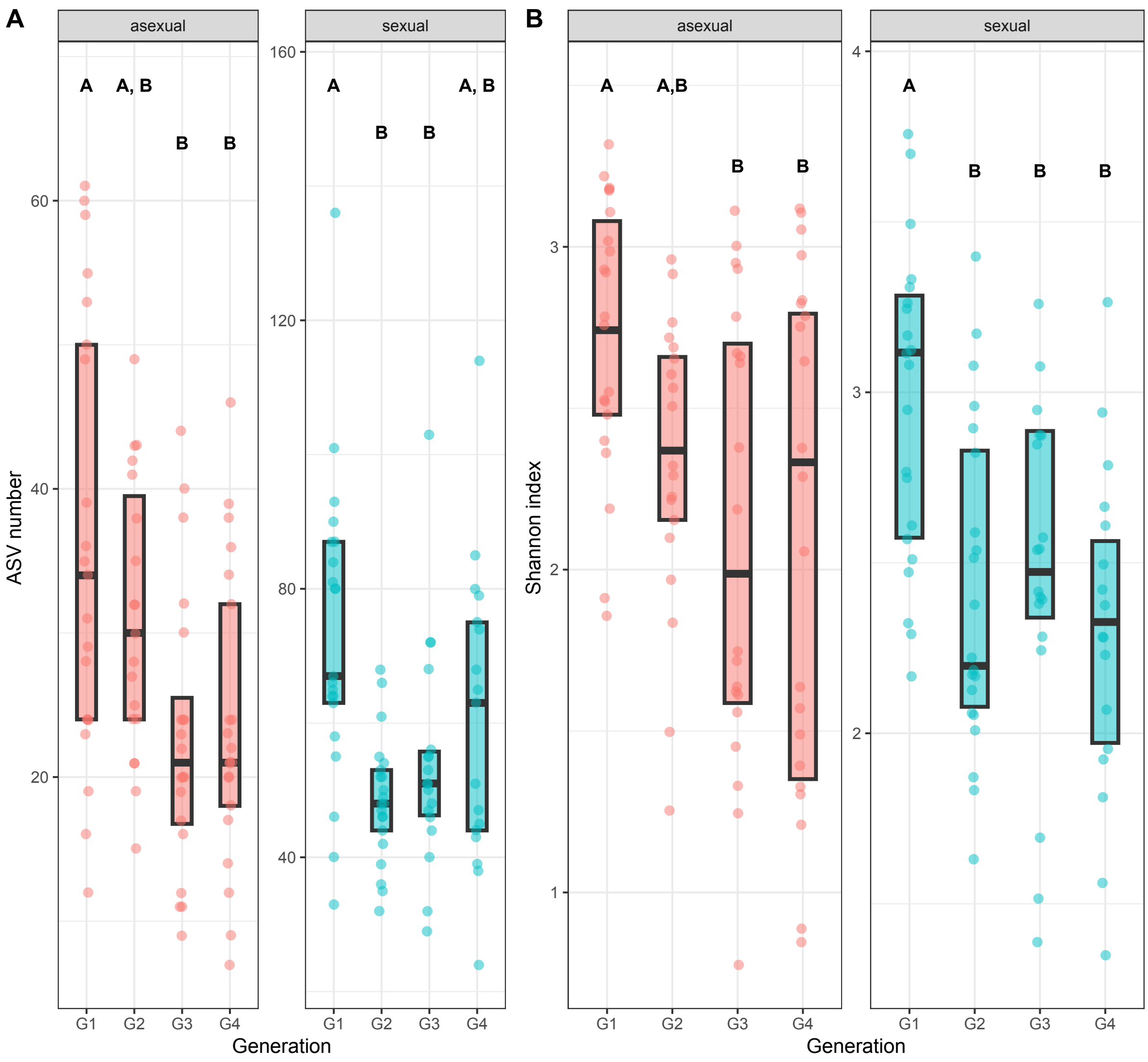

Figure S3: Alpha diversity expressed as **A)** ASV number and **B)** Shannon index of asexual (*Wolbachia*-infected) and sexual (*Wolbachia*-uninfected) *A. japonica* wasps reared in seven replicated lines over four generations (G1 to G4) in the laboratory after removal of *Wolbachia* reads from the data. Boxplots show the median and interquartile range. Letters above boxes indicate significant differences between generations.

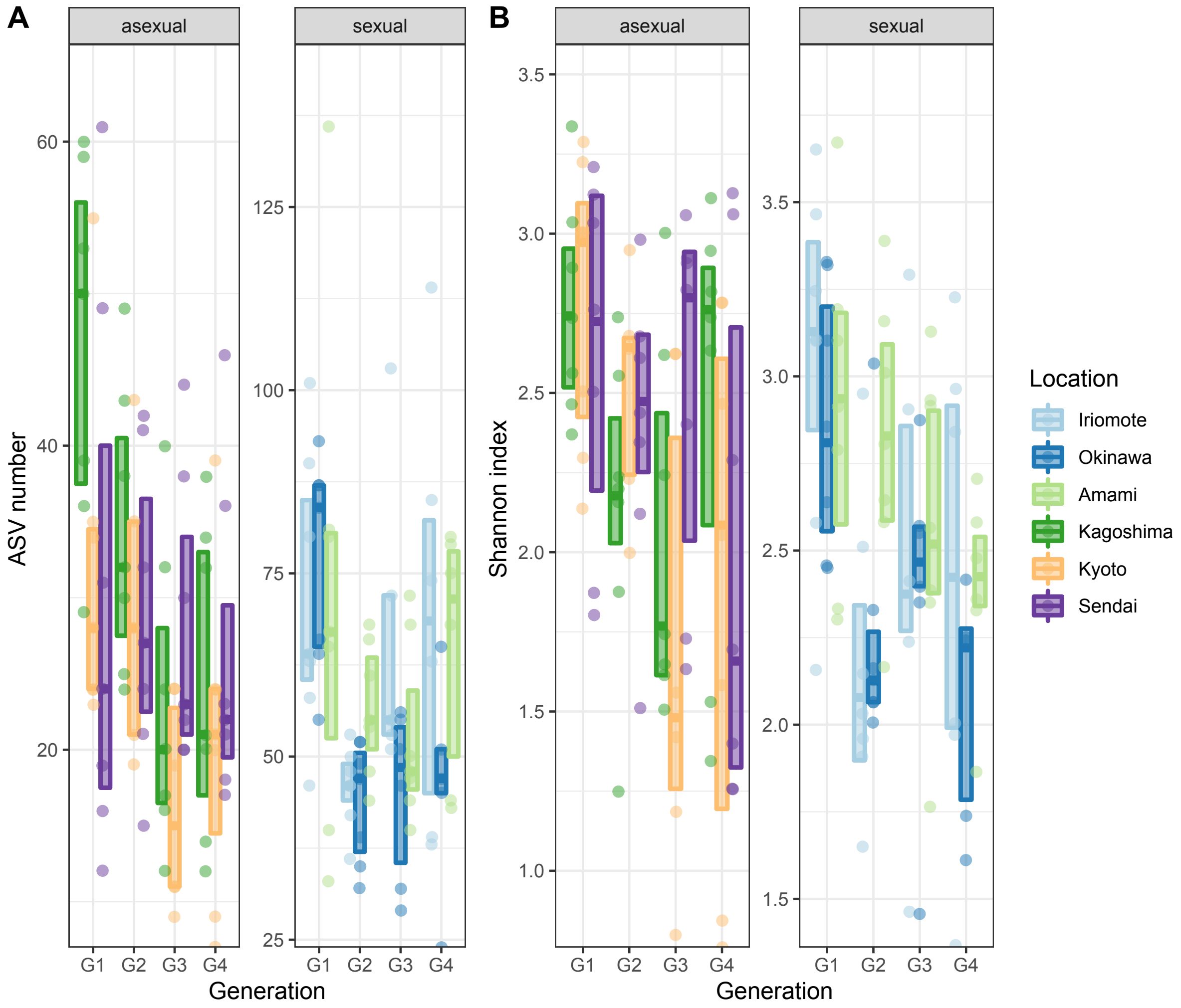

Figure S4: Alpha diversity expressed as **A)** ASV number and **B)** Shannon Index of seven replicated lines of three asexual, *Wolbachia*-infected (Kagoshima, Kyoto, Sendai) and three sexual, *Wolbachia*-uninfected lines (Iriomote, Okinawa, Amami) over four generations (G1 to G4) in the laboratory after removal of *Wolbachia* reads from the data. Only the location Okinawa showed statistically significant differences over generations (see Table S1). Boxplots show the median and interquartile range.

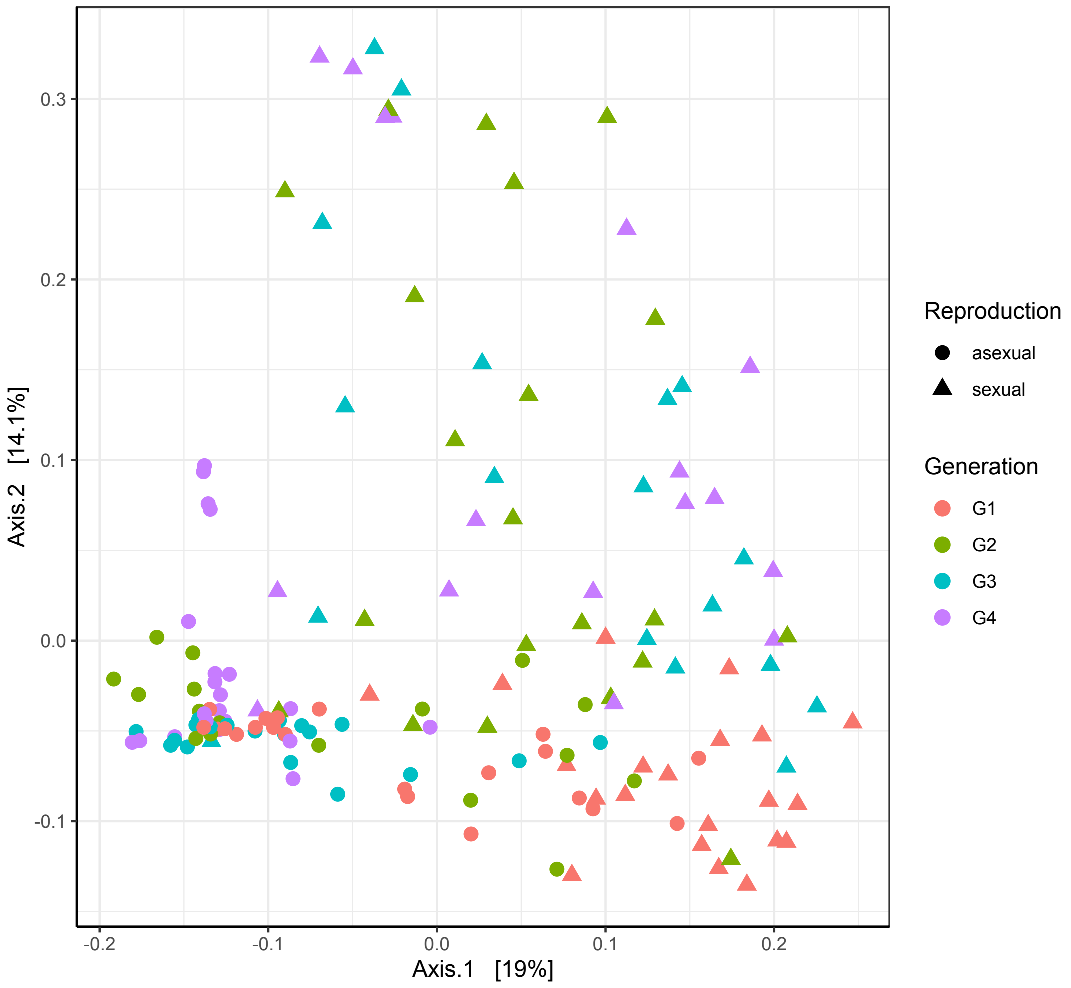

Figure S5: Bacterial community composition after removal of *Wolbachia* between the two reproductive modes, sexuals (dots) and asexuals (triangles), of *A. japonica* over four generations of laboratory culturing. Colours indicate locations and shapes generations.

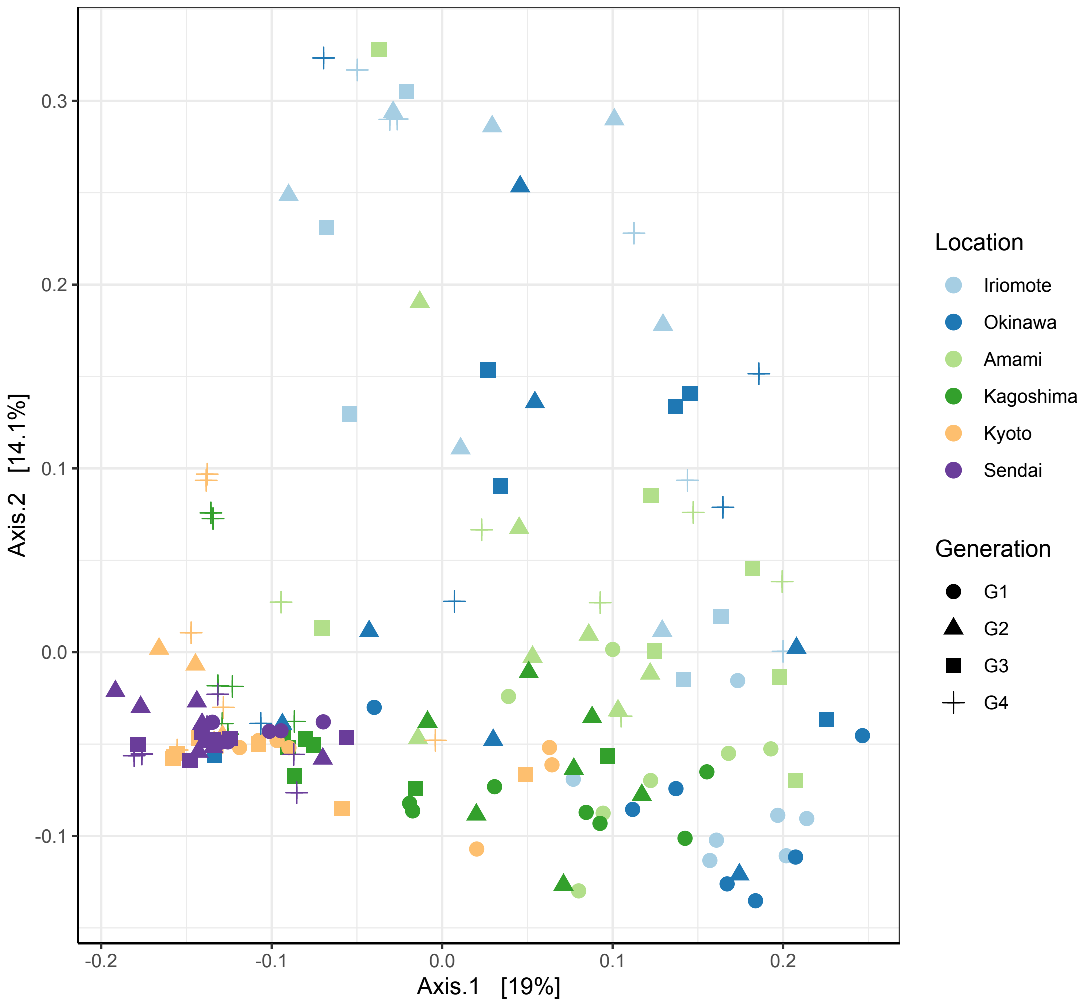

Figure S6: Bacterial community composition for the six tested lines of the wasp *A. japonica* over four generations of laboratory culturing after the removal of *Wolbachia*. Colours indicate locations, and shapes indicate generations.

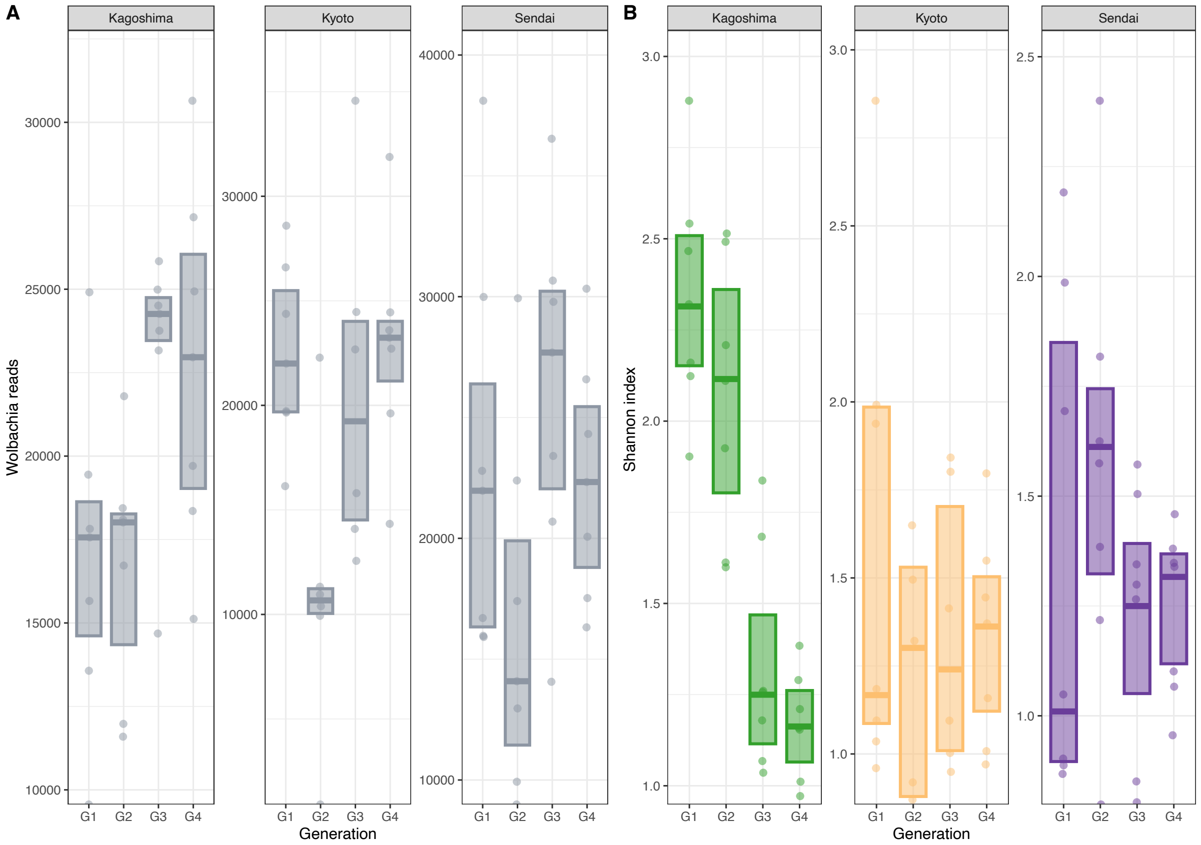

Figure S7: **A)** Abundance of *Wolbachia* cells measured as the number of *Wolbachia* reads over four generations (G1-4) from three asexual *A. japonica* lines. Reads extracted out of the unrarefied but normalised dataset are plotted. **B)** Alpha diversity expressed as Shannon Index of seven replicates from three asexual, *Wolbachia*-infected *A. japonica* lines (Kagoshima, Kyoto, Sendai) over four generations (G1 to G4) in the laboratory. Boxplots show the median and interquartile range. Letters above boxes indicate significant differences between generations.
